# Inflammation clinical trial in mild to moderate Alzheimer's disease: TSPO‐PET results

**DOI:** 10.1002/alz70856_103463

**Published:** 2025-12-24

**Authors:** Belen Pascual, Alireza Faridar, Quentin Finn, Nazaret Gamez, Stanley H Appel, Joseph C. Masdeu

**Affiliations:** ^1^ Houston Methodist Research Institute, Houston, TX, USA

## Abstract

**Background:**

Brain inflammation can be assessed using translocator protein (TSPO) PET because TSPO is highly expressed by microglia, peripherally‐derived macrophages, and astrocytes. However, TSPO PET cannot differentiate between M1 (neurotoxic) and M2 (neuroprotective) microglial phenotypes. The utility of TSPO PET in tracking brain inflammation during Alzheimer's disease (AD) clinical trials targeting inflammation remains largely unexplored. To address this gap, we report on a TSPO‐PET study performed before and after an immunomodulation clinical trial for AD. TSPO‐PET was performed using ^11^C‐ER176, a tracer with high specific binding, a favorable metabolite profile, and increased uptake in AD, particularly in brain regions with elevated tau levels.

**Method:**

Thirty‐eight AD patients (ages 50‐86, MMSE scores 12‐26) participated in a phase 2a, randomized, double‐blind, placebo‐controlled trial evaluating two dosing frequencies of Interleukin‐2 (IL‐2). Over a 21‐week period, 9 participants received IL‐2 every 4 weeks (IL‐2 q4wks), 10 received IL‐2 every 2 weeks (IL‐2 q2wks), and 19 received placebo. ^11^C‐ER176 PET (using standard methods and an arterial‐input function), blood/CSF biomarkers, and clinical scores were obtained before and after treatment.

**Result:**

Group analyses revealed no differences in TSPO‐PET binding before and after the IL‐2 dosing regimens or placebo. Both IL‐2 regimens significantly increased Treg numbers and function, with IL‐2 q4wks being more effective in enhancing Treg counts and Foxp3 mean fluorescence intensity. In the IL‐2 q4wks group, CSF Aβ42 levels improved significantly (*p* = 0.045), with a strong trend toward stabilized CSF NfL levels (*p* = 0.061). CSF GFAP levels remained stable with both IL‐2 q4wks and IL‐2 q2wks regimens but increased substantially in the placebo group. No significant changes were observed in CSF *p*‐Tau181 levels across all groups. Notably, IL‐2 q4wks regimen was associated with a slowing of cognitive decline compared to placebo, highlighting their potential therapeutic benefit in this context.

**Conclusion:**

While IL‐2 immunotherapy every 4 weeks enhanced Treg populations, showing promising trends in AD biomarkers and clinical scales, TSPO‐PET was largely unchanged over the 21 weeks of the trial. A longer follow‐up period, a larger sample size, or, more likely, PET tracers specific for M1 and M2 macrophages or other inflammation targets are needed for future clinical trials.

